# Data-Driven Approaches Used for Compound Library Design for the Treatment of Parkinson’s Disease

**DOI:** 10.3390/ijms24021134

**Published:** 2023-01-06

**Authors:** Oscar Barrera-Vazquez, Jose Alberto Santiago-de-la-Cruz, Nadia Alejandra Rivero-Segura, Edgar Antonio Estrella-Parra, Genaro Salvador Morales-Paoli, Edgar Flores-Soto, Juan Carlos Gomez-Verjan

**Affiliations:** 1 Departamento de Farmacología, Facultad de Medicina, Universidad Nacional Autónoma de México, Mexico City 04510, Mexico; 2 Dirección de Investigación, Instituto Nacional de Geriatría (INGER), Mexico City 10200, Mexico; 3 Posgrado de Ciencias Genómicas, Universidad Autónoma de la Ciudad de México, San Lorenzo 290, Colonia Del Valle Sur, Benito Juarez, Mexico City 03100, Mexico; 4 Laboratorio de Fitoquímica, UBIPRO, FES-Iztacala, Universidad Nacional Autónoma de Mexico, Av. De los Barrios No.1, Los Reyes Iztacala, Tlalnepantla 54090, Mexico; 5 Campus en Línea, Universidad Tecnológica de México-UNITEC, Mexico City 09810, Mexico

**Keywords:** data-driven approach, chemoinformatics, Parkinson’s disease, computational drug design

## Abstract

Parkinson’s disease (PD) is the second most common neurodegenerative disease in older individuals worldwide. Pharmacological treatment for such a disease consists of drugs such as monoamine oxidase B (MAO-B) inhibitors to increase dopamine concentration in the brain. However, such drugs have adverse reactions that limit their use for extended periods; thus, the design of less toxic and more efficient compounds may be explored. In this context, cheminformatics and computational chemistry have recently contributed to developing new drugs and the search for new therapeutic targets. Therefore, through a data-driven approach, we used cheminformatic tools to find and optimize novel compounds with pharmacological activity against MAO-B for treating PD. First, we retrieved from the literature 3316 original articles published between 2015–2021 that experimentally tested 215 natural compounds against PD. From such compounds, we built a pharmacological network that showed rosmarinic acid, chrysin, naringenin, and cordycepin as the most connected nodes of the network. From such compounds, we performed fingerprinting analysis and developed evolutionary libraries to obtain novel derived structures. We filtered these compounds through a docking test against MAO-B and obtained five derived compounds with higher affinity and lead likeness potential. Then we evaluated its antioxidant and pharmacokinetic potential through a docking analysis (NADPH oxidase and CYP450) and physiologically-based pharmacokinetic (PBPK modeling). Interestingly, only one compound showed dual activity (antioxidant and MAO-B inhibitors) and pharmacokinetic potential to be considered a possible candidate for PD treatment and further experimental analysis.

## 1. Introduction

Parkinson’s disease (PD) is the second most common neurodegenerative disease in older individuals; it is characterized by neuronal loss in the substantia nigra leading to a significant decrease in the striatal dopamine, and the simultaneous display of bradykinesia, motor features, such as tremors and rigidity, as well as non-motor symptoms such as cognitive decline, depression, and pain [1].

At the molecular level, PD has been described as involving α-synuclein aggregation, proteostasis imbalance, mitochondrial dysfunction, oxidative stress, excitotoxicity, impaired axonal transport, and neuroinflammation, suggesting such mechanisms as potential targets to content against neuronal loss in PD [2,3]. Currently, the canonical drug treatments in PD are palliatives that slow down the disease progression providing relief from the severe symptoms of the disease [2,4,5]. Moreover, the BBB is a highly selective barrier that separates the central nervous system (CNS) from systemic circulation. Thus, the BBB also represents another obstacle to the drug discovery against PD since the BBB integrity seems to remain intact, at least in the early stages of the disease; thus, all the novel therapeutic strategies may be guided by the notion that the BBB is impermeable to several compounds [6].

In this context, cheminformatics provides computational methods that improve the process of predicting chemical compounds’ physicochemical and biological properties on a scale unattainable by traditional methods. Even they can predict whether a compound may cross the BBB since the advancement in computational modeling has developed a tool to predict the BBB permeability of drugs according to their chemical and physicochemical properties [7,8]. Besides, major pharmaceutical companies use these tools in the early phases of drug design [9]; their results have several applications in developing novel compounds to treat HIV, diabetes, cancer, and other diseases [9,10,11,12]. Additionally, physiologically-based pharmacokinetic (PBPK) models are novel mechanistic models that transcribe anatomical, physiological, physical, and chemical descriptions of the phenomena involved in the complex ADME (absorption, distribution, metabolism, and excretion) process. In this sense, PBPK models may have purely predictive uses for both toxicity risk assessment and therapeutic drug development [13].

Interestingly, there are few cheminformatic studies on PD, one of them carried out the design and synthesis of MAO-B inhibitors by using 3-D cedrane scaffold analysis [14,15], showing selective activity for markers related to α-tubulin, mitochondria, and lysosome on PD patient-derived cell line. In this sense, MAO-B inhibitors (MAOB-I) such as gamma-decanolactone or deprenyl have been helpful as a specific group of drugs against PD since such enzyme increases H_2_O_2_ concentration via the interaction of mitochondrial complex I [16,17]. Interestingly, although oxidative stress plays a crucial role in the degeneration of neurons in PD, most MAOB-I have not been tested or designed for its antioxidant activity [18,19].

Therefore, in the present study, we aim to rationally design more efficient compounds with activity against MAO-B and oxidative stress. Using experimental data of natural products published between 2015–2021 against PD, we built a pharmacological network to obtain the most connected nodes (compounds) and use its structure to obtain a novel library of compounds. Subsequently, the library was filtered with a test against MAO-B. From the most active compounds, we also tested their antioxidant and pharmacokinetic potential by molecular docking and PBPK modeling.

## 2. Results

### 2.1. Compounds and Pharmacological Targets against PD

**Figure 1:**
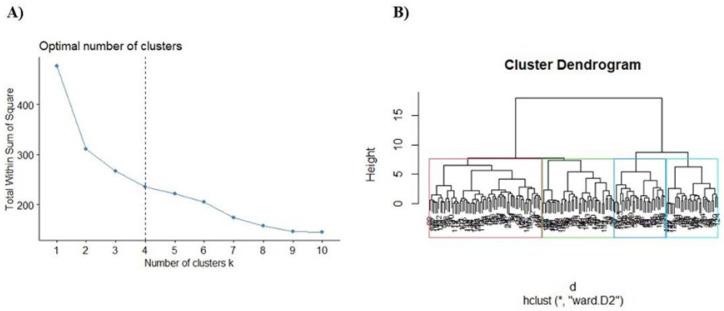
Selection of the most representative compounds for the treatment of PD. (**A**) Estimation of the optimal number of clusters. (**B**) Dendrogram−hierarchical clustering (hclust) of the compounds for PD treatment using Ward’s method (*, ward D2).

Two hundred fifteen compounds were retrieved from Science Direct and Scopus to build a database including the name compounds, chemical structure, SMILE (*simplified molecular-input line-entry system*), the experimental model tested, and their molecular targets (Table S1 in the Supplementary Materials). From this database, we also calculated the molecular descriptors with DataWarrior software. After determining the fingerprints, the elbow method was used to obtain the number of clusters for the hierarchical clustering analysis (Figure 1A), which showed an *n* = 4 as the most appropriate cluster number for our analysis. Then we build a hierarchical clustering (dendrogram) to obtain the most relevant compounds through their structural similarity (Figure 1B).

### 2.2. A Network between Drugs and Targets for Parkinson’s

**Figure 2:**
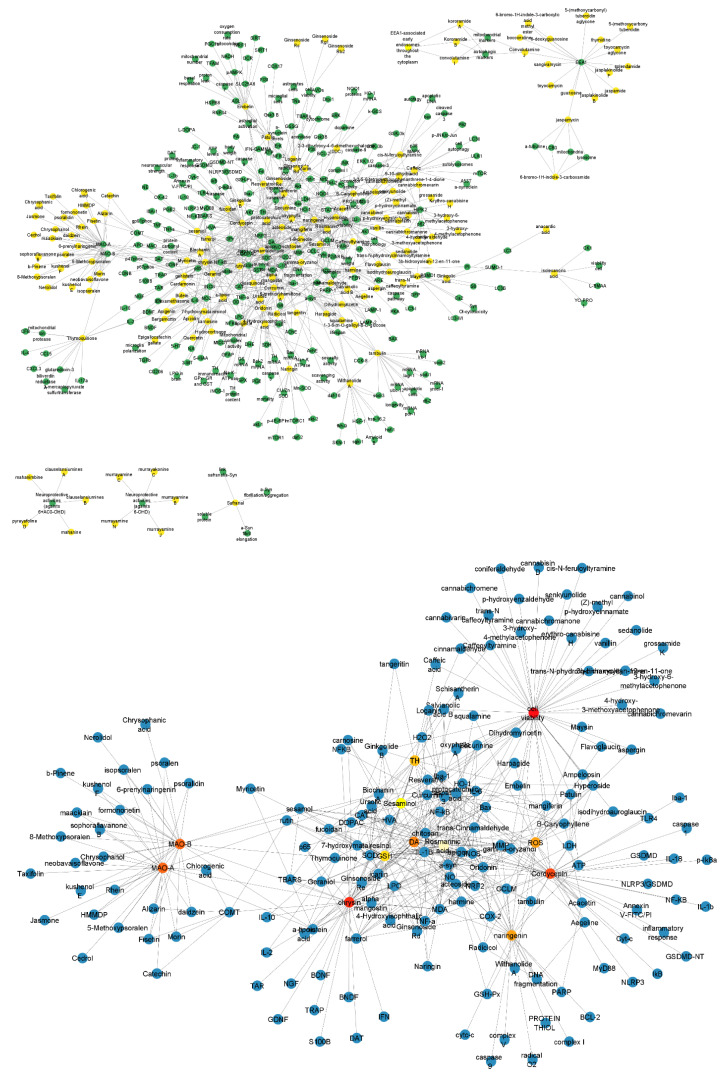
Structural network of the drugs used for the PD treatment and their pharmacological targets and their representative components. The upper structural network organizes the compounds (green nodes) and their pharmacological targets (yellow nodes). The structural network below derived from the Cytohubba plug-in shows the most connected nodes (rosmarinic acid, chrysin, naringenin, and cordycepin, and MAO-A and MAO-B). Nodes represent the compounds and pharmacological targets, and edges are the reported interactions between them.

Using the data from Figure 1B, we built a structural network that represents the compounds for the treatment of PD (green nodes) and their pharmacological targets (yellow nodes) (Figure 2). Then, we apply the Cytohubba plug-in to identify the most connected node in the network (Figure 2). We identified that the most connected compounds from this network were rosmarinic acid, chrysin, naringenin, and cordycepin. The most representative targets were monoamine oxidase type A (MAO-A) and MAO-B [20].

### 2.3. Bemis-Murcko Scaffold Analysis

**Figure 3:**
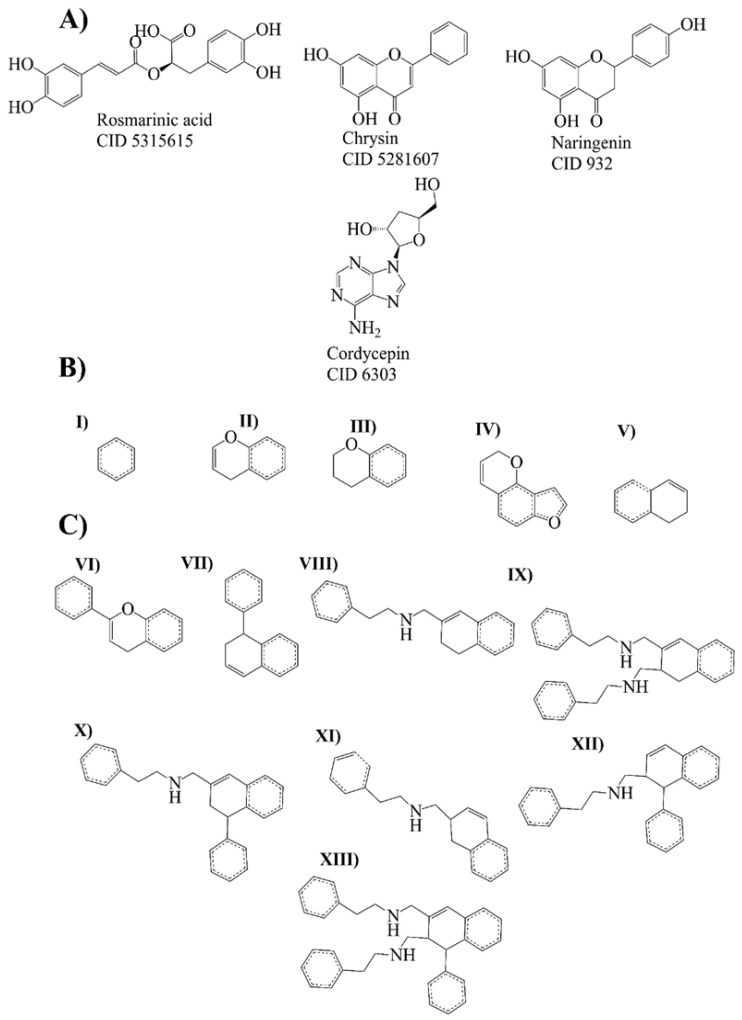
Molecular leaders and evolutionary library. (**A**) Molecule leaders for the library: rosmarinic acid, chrysin, naringenin, and cordycepin. (**B**) Bemis–Murcko fragments (strings) from rosmarinic acid, chrysin, naringenin (I–IV), and cordycepin (V). (**C**) Bemis–Murcko fragments (frameworks) from rosmarinic acid, chrysin, naringenin (VI), and cordycepin compounds (VII–XIII).

Bemis–Murcko scaffold analysis was performed to estimate the ability of the rosmarinic acid, chrysin, naringenin, and cordycepin to generate new scaffolds. The fragments are shown as strings and frameworks representing the union of ring systems and linkers in a molecule, side chains, and linkers [21]. At the end of the analysis, ten strings and ten frameworks related to rosmarinic acid, chrysin, naringenin, and cordycepin were obtained (Figure 3).

### 2.4. Evolutionary Library and Docking with Targets of PD

An evolutionary library was built to create new compounds using structures and fragments (Bemis–Murcko scaffold analysis) from the rosmarinic acid, chrysin, naringenin, and cordycepin (Figure 3A–C). We obtained 22 structures derived directly from compounds and 26 derived compounds for the fragments (supplementary data).

**Figure 4:**
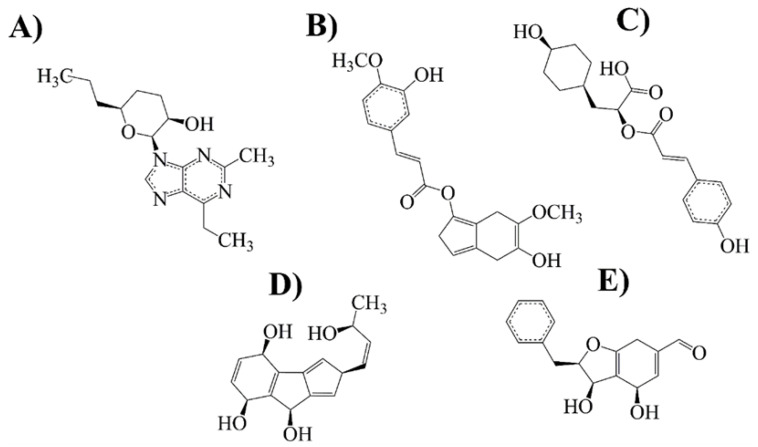
Selected compounds with more conformational stability and biological activity on MAO-B. (**A**) Cordycepin-derived compound (21 NP); (**B**) rosmarinic acid-derived compound (14 NP); (**C**) rosmarinic acid-derived compound (6 PP); (**D**) naringenin-derived compound (20 NP) and (**E**) chrysin derived compound (22 NP).

Then, we proceeded to carry out a docking assay between MAO-B and compounds of the library; as a control for MAO-B binding, we used selegiline and rasagiline, which have been widely described as potent inhibitors of this target. Results from Table 1 and Figure 4 showed that 18 compounds interacted with the binding site of MAO-B, and only five compounds achieved the most stable conformation against the MAO-B.

### 2.5. Drug-Likeness and Lead-Likeness Analysis

We evaluated drug-likeness and pharmacokinetic parameters from the previous compounds (21 NP, 14 NP, 6 PP, 20 NP, and 22 NP). All these compounds satisfy Lipinski’s rule of five, and only compound 14 NP has a violation of lead-likeness by its molecular weight (MW > 350) (Supplementary Materials). Nevertheless, the five compounds have pharmacokinetic parameters necessary to be considered drug-likeness. The SwissADME Synthetic Accessibility (SA) score for the five selected compounds presents a value between 3–5, meaning that these compounds show an intermediate difficulty in their chemical synthesis. Lastly, a prediction *in silico* of toxicological properties was performed for these compounds. They do not present mutagenic, tumorigenic, or reproductive efficacy effects.

### 2.6. Antioxidant Activity against P450 and NO

**Figure 5:**
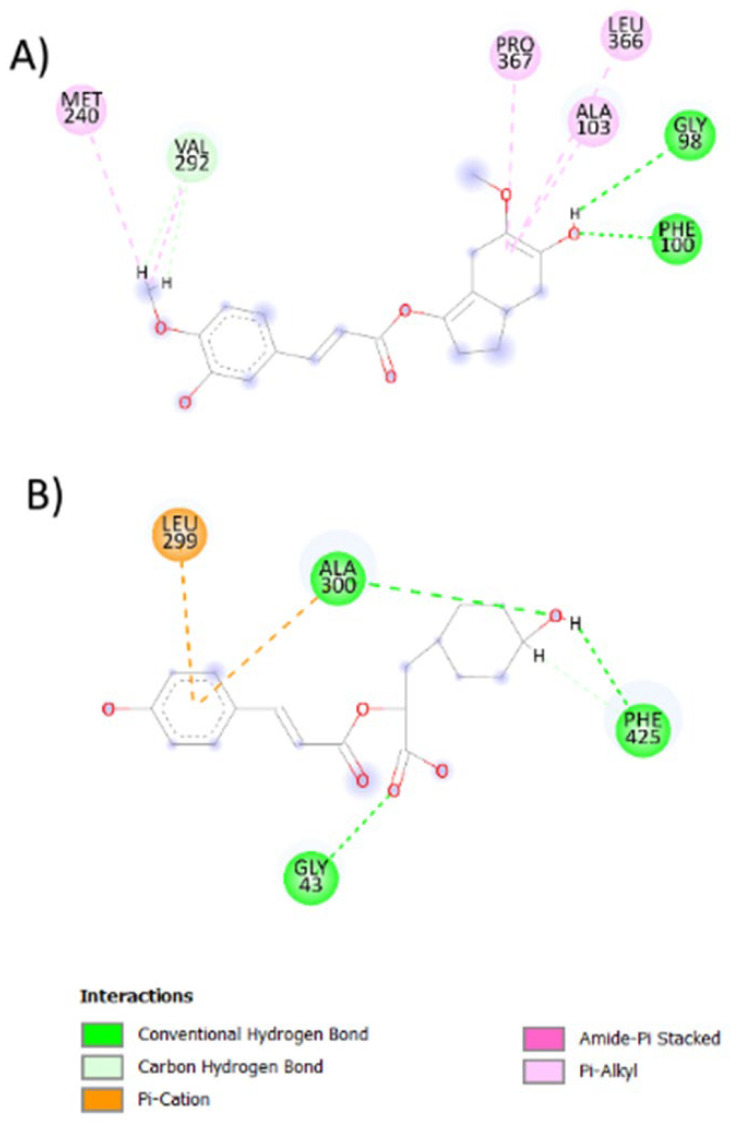
2D representation of the best-coupled interactions. (**A**) Interaction of CYP450 with rosmarinic acid-derived compound (14 NP, ΔG = −8.617973). (**B**) Interaction of NO with rosmarinic acid-derived compound (6 APP, ΔG = −8.725966).

Since one of the main molecular problems in PD is oxidative stress, we aim to evaluate whether the mentioned molecules possess antioxidant activity. As results from the molecular docking, we obtain that compound 14NP show more affinity for CYP450 (ΔG = −8.617973) while compound 6 APP interacts preferably with NADPH oxidase (NO) (ΔG = −8.725966) (Table 2 and Figure 5).

### 2.7. Pharmacokinetic Prediction (PBPK Modeling)

**Figure 6:**
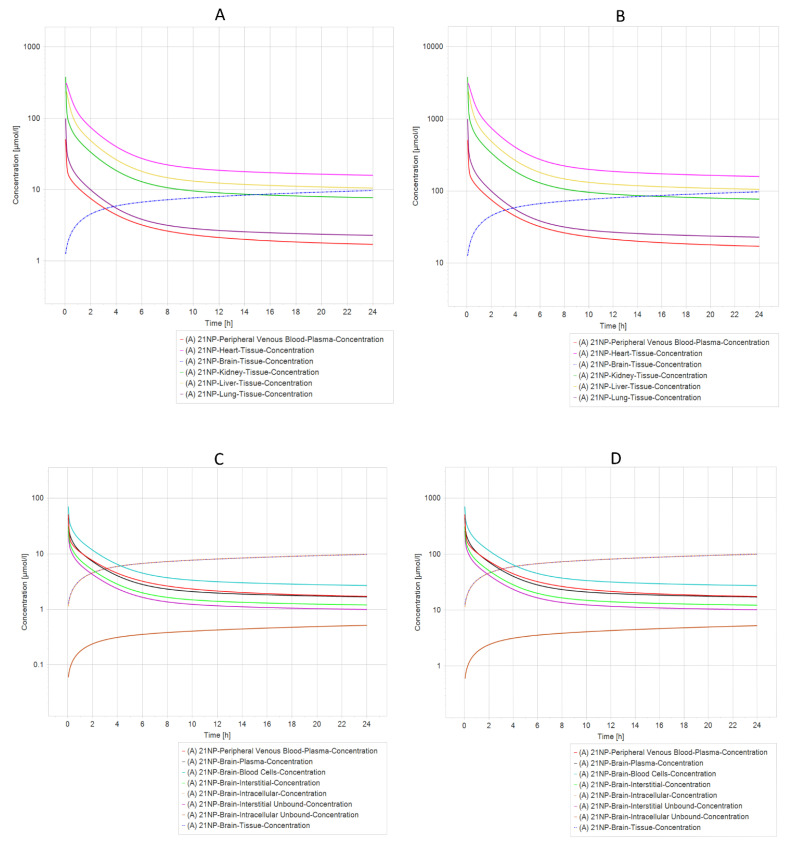
PBPK comparison of different model simulations across different dosages of 21NP molecule modeled in a healthy patient. (**A**) Five different compartments (organs) dosed with 10 mg/kg in a healthy individual. (**B**) Five different compartments (organs) dosed with 100 mg/kg in a healthy individual. (**C**) Different brain compartments dosed with 10 mg/kg in a healthy individual. (**D**) Different brain compartments dosed 100 mg/kg in a healthy individual. The heart, liver, and kidney are the organs with the longest elimination time of molecule 21 NP, whereas in the Brain (**C**,**D**), there is an increased concentration of molecule 21 NP in tissue and intracellular compartments.

**Figure 7:**
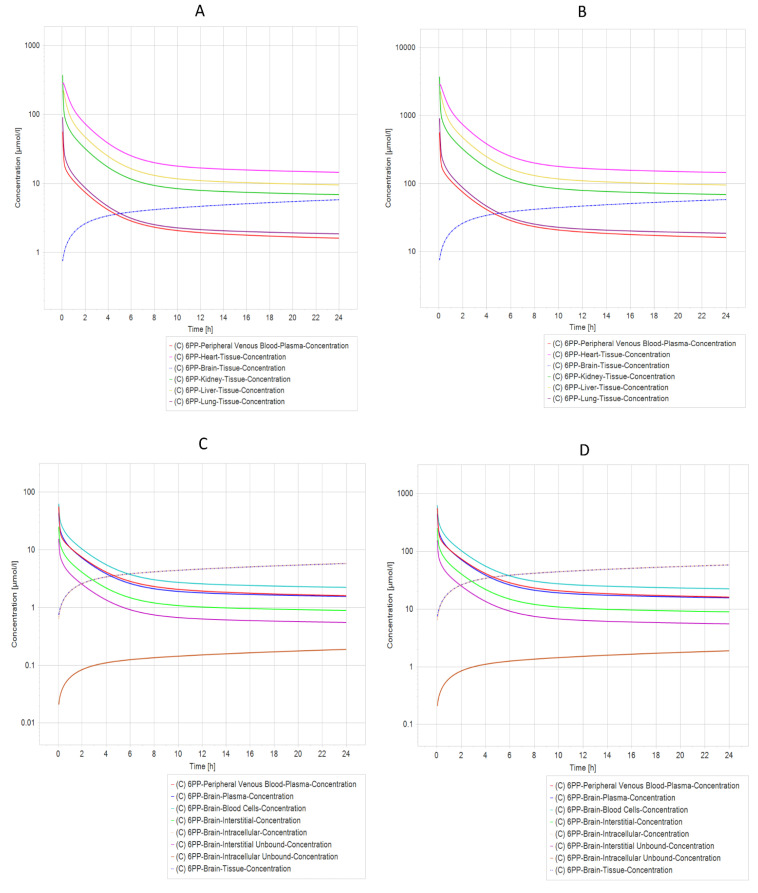
PBPK Model comparison of different model simulations across different dosages of 6 APP molecules modeled in a healthy individual. (**A**) Five different compartments (organs) dosed with 10 mg/kg in a healthy individual. (**B**) Five different compartments dosed with 100 mg/kg in a healthy individual. (**C**) Different brain compartments dosed with 10 mg/kg in a healthy individual. (**D**) Different brain compartments dosed 100 mg/kg in a healthy individual. The heart, liver, and kidney are the organs that have the longest elimination time of molecule 6 APP, whereas (**C**,**D**) (Brain) have an increase in the concentration of molecule 6 APP in tissue and intracellular compartments.

Another exciting result from PBPK simulation concluded that compounds 21 NP (Figure 6A,B) and 6 PP (Figure 7A,B) were the most relevant due to their pharmacokinetic curves. The half-life analysis shows us that 21 NP and 6 PP compounds are up to 75 h in the heart, kidney, liver, and lung. In comparison, 20 NP and 22 NP are over 3000 to 4000 h, respectively, in the same organs. One of the main concerns in drug design is the delivery to the brain. Interestingly, both compounds (21 NP and 6 PP) demonstrate to cross the BBB efficiently since when 21 NP is administered at 10 mg/Kg or 100 mg/Kg, the concentration in the brain is 9.76 µmol/L or 97.6 µmol/L, respectively (Figure 6C,D). Moreover, the 6PP compound that crosses the BBB reaches concentrations in the brain around 5.82 µmol/L and 58.16 µmol/L when administered at 10 mg/Kg or 100 mg/Kg, respectively (Figure 7C,D). Complete results of the parameters, predicted by PBPK modeling in both compounds, are shown in Supplementary Materials Tables S2–S5.

## 3. Discussion

As mentioned above, PD is one of the most prevalent neurodegenerative diseases in the aged population. Natural products have been recognized as the primary source for discovering novel potential therapeutic drugs for many neurodegenerative diseases such as PD. The development of cheminformatics tools has improved drug discovery daily and offers a wide range of tools that may be more efficient in decreasing the side effects and improving the patient’s quality of life [11,19,22,23]. Additionally, many authors mention that BBB permeability is a significant obstacle to drug discovery. For instance, it has been reported that despite the promising results demonstrated by the humanized monoclonal antibodies against α-synuclein, less than 1% of the antibody crosses the BBB [24]. Hence, developing more efficient drugs against PD and other diseases from the CNS may consider the BBB permeability. In this context, in this study, we aim to rationally design more efficient compounds with activity against MAO-B and oxidative stress, using experimental data of natural products published between 2015–2021 against PD. First, we built a pharmacological network to obtain the most connected nodes (compounds) and used its structure to obtain a novel library of compounds. Subsequently, the library was filtered with a test against MAO-B. From the most active compounds, we also tested their antioxidant and pharmacokinetic potential by molecular docking and PBPK modeling. The results demonstrate that rosmarinic acid, chrysin, naringenin, and cordycepin compounds were the most suitable for drug design.

Cordycepin has a vast spectrum of bioactive properties, such as anti-inflammatory and antidepressants [25,26,27,28]. In animal models of PD, cordycepin induces anti-inflammatory and neuroprotective effects via mitochondrial fission regulation [29]. At the same time, in another study, it has been reported that cordycepin ameliorates locomotor impairments, inhibits the activation of the NLRP3 inflammasome, inhibits the TLR/NFkB pathway. and suppresses the pyroptosis and inflammatory cascades [30,31]. Similarly, romantic acid improves motor function and reduces proinflammatory cytokines in PD animal models [32]. Also, rosmarinic acid alleviates neuroinflammation, microglial activation, and apoptosis by regulating the miR-155-5p [33].

Meanwhile, naringenin has broad biological effects such as anti-viral and anti-aging [34,35,36]. Interestingly, rodent models of PD reported that naringenin decreases dopaminergic degeneration by regulating α-synuclein pathology, neuroinflammation and oxidative stress [37,38]. These data support our findings and suggest the potential use of naringenin derivatives in vivo PD models. Chrysin in animal models of PD induced with 6-hydroxydopamine also increases proinflammatory cytokines and decreases dopamine and homoallylic acid levels [39,40,41,42,43]. Interestingly, the treatment with chrysin induces neuroprotection by decreasing neuroinflammatory markers and increasing levels of brain-derived neurotrophic factor (BDNF) recovering dopaminergic neurons in the striatum [44].

The previously mentioned leader molecules were subjected to the Bemis–Murcko framework and string analysis [45,46,47], from which a series of compounds were obtained and filtered by a docking test with MAO-B [48]. A double-cavity active site characterizes MAO-B, and its conformation is ligand-dependent [49]. Regarding the compounds selected for the docking test, only eighteen interact significantly with MAO-B, some with greater affinity than selegiline or rasagiline, currently used in clinics as MAOB-I. The compounds with more stability (ΔG) [50] toward MAO-B were 21 NP, 14 NP, 6 PP, 20 NP, and 22 NP. Moreover, the results from the docking analysis (estimated ΔG and binding energy) suggest that compound 21 NP is the most stable and thermodynamically favorable for binding to the MAO-B active site, suggesting that such a compound should be more effective for PD treatment.

Additionally, docking assays with the antioxidant enzymes (CYP450 and NO [51]) showed that compounds 14 NP and 6 PP have the highest conformational stability. These data indicate that the ligand-protein interaction possibly affects the function of these proteins and therefore generates an antioxidant activity. However, experimental phase analyses must be performed to validate such findings. Interestingly, these results align with others that aim to explore whether natural product-like caffeine derivates are potential inhibitors of MAO-B and antagonists of the adenosine receptor A_2A_, suggesting that virtual screening provides valuable insights into developing novel antiparkinsonian drugs in an affordable way [52]. Additionally, our results demonstrate that the interaction between both compounds (21 NP and 6 PP) is stabilized by hydrogen bonds that have been reported to have fewer adverse secondary effects than those compounds that interact with MAO-B by covalent bonds.

On the other hand, PBPK modeling and simulation approaches have gained popularity in recent years, particularly for predicting the impact of drug-drug interactions, selecting an optimal dose and clinical trial design for geriatric applications, and characterizing the impact of organ impairment [53]. Our PBPK simulation found that only 21 NP and 6 PP have acceptable pharmacokinetic values for their potential use. Interestingly, both compounds cross the BBB and allocate intracellular compartments in the brain, suggesting that they may be potential drugs with efficient effects against PD. Nevertheless, it is essential to mention that experimental studies should be performed to validate such results.

## 4. Materials and Methods

### 4.1. Data Curation

Data were retrieved from two databases, Scopus and Science Direct, both accessed on 13 May 2021. We used the keywords “Natural Products” and “Parkinson’s Disease”. As a result, we obtained a total of 336 articles (2815 from Scopus and 501 from Science Direct Databases). From there, we only included the articles that meet the following inclusion criteria: only original articles that test the compounds experimentally, published in English between 2015–2020, and with available data accessibility. Since the database was manually curated, we avoided duplicated articles or miscues. Finally, we obtained only 85 articles from which we extracted 215 molecules for further analysis.

### 4.2. Structural Network Analysis

Structural network analyses were built using the curated databases described above using the Cytoscape (software 3.8.2 version, https://cytoscape.org/ accessed on 21 October 2022) [54] (Supplementary Materials). Later, a sub-network was constructed identifying the most connected nodes using the Cytohubba plug-in [55].

### 4.3. Fingerprints Analysis

For fingerprint analysis of NPs (Natural Products), we used ChemmineR and rcdk in RStudio (software version 3.4 https://posit.co/ accessed on 20 June 2022) [56]. The SDF files required for this software were obtained from PubChem Database (https://pubchem.ncbi.nlm.nih.gov/) accessed on 20 June 2021. The fingerprint analysis used a symbolic value with a default length of 1024 (number of bits), and then we performed a clusterization by using the Tanimoto coefficient to compare the structural similarity of the drugs. Later, the elbow method was used to determine the optimal number of clusters. The molecules were classified into four groups using as a parameter the molecular distances estimated by Ward’s clustering method, which is the most popular hierarchical clustering algorithm used in drug discovery [56].

### 4.4. Bemis Murcko Scaffold Analysis

The Bemis–Murcko fragments were generated using the rcdk package from RStudio software version 3.4. using the structures of the compounds obtained by fingerprint and structure network analyses [56]. The structures of the compounds were obtained from PubChem Database (https://pubchem.ncbi.nlm.nih.gov/ accessed on 20 June 2021).

### 4.5. Evolutionary Library Construction

An evolutionary library was constructed using Osiris Datawarrior (Software version 5.5.0-2021, https://openmolecules.org/datawarrior/index.html accessed on 20 June 2021) [54]. The Bemis–Murcko fragments and the compounds were obtained from fingerprint and structural network analysis. The fitness criteria used to construct this evolutionary library were: molecular weight (Da), octanol–water partition coefficient (AlogP), the number of H-acceptors (HBA), the number of H-donors (HBD), the number of rotatable bonds, total polar surface area (TPSA), and aromatic-bound, accounted for components of the Quantification of the Estimation of Druglikeness (QED), due to this index is the most suitable for the drug-likeness determination over the compounds [57]. The generation criteria were automatic generations, 128 compounds per generation, and 20 compounds survive a generation.

### 4.6. Docking Modeling

Once we chose compounds from the previously mentioned library, blind docking was performed since this is the most commonly used and accepted type of modeling for drug discovery. We used the Swissdock web server (http://www.swissdock.ch, accessed on 20 June 2022), which predicts the binding modes between different targets and the ligands. A complete description of the algorithm used is in [58]. We tested the target involved in PD: The X-ray crystal structure of MAO-B (PDB accession number: 4A79 [59]) was downloaded from the Protein Data Bank database. MAO-B consists of a two-domain molecular architecture. Each identical monomer consists of 520 amino acids. This study used one monomer (chain A) previously described [59]. The protein was prepared by adding hydrogens, adjusting bond orders, proper ionization states, and refining overlapping atoms with Chimera (software version 1.16) USCF Chimera (https://www.cgl.ucsf.edu/chimera) accessed on 20 June 2022 [60]. Subsequently, the selected ligands (cordycepin-derived compound 21 NP, rosmarinic acid-derived compound 14 NP, rosmarinic acid-derived compound 6 PP, naringenin-derived-compound 20 NP, chrysin-derived compound 22 NP, chrysin-derived compound 20 PP, rosmarinic acid- derived compound 18 NP, chrysin-derived compound 19 NP, naringenin-derived compound 18 NP, chrysin-derived compound 11 PP, chrysin fragment 16 NP, chrysin fragment 20 NP, naringenin fragment 19 PP, rosmarinic acid fragment 15 NP, rosmarinic acid fragment 21 NP, rosmarinic acid fragment 16 PP, rosmarinic acid-derived compound 19 PP, and naringenin fragment 23 NP).

### 4.7. Drug-Likeness and Lead-Likeness Analysis

Structures of compounds were analyzed with the web tool SwissADME (http://www.swissadme.ch, accessed on 20 June 2022) to evaluate drug-likeness, synthetic accessibility, and pharmacokinetic parameters [61].

### 4.8. In Silico Prediction of Toxicity

Structures of compounds were analyzed with Osiris Datawarrior (Software version V5.5.0-2021, https://openmolecules.org/datawarrior/, accessed on 20 June 2021) [45] to predict drug-likeness, drug scores, and four toxicity risks: mutagenicity, tumorigenicity, reproductive effectiveness, and irritant.

### 4.9. In Silico Antioxidant Activity

The antioxidant activity of compounds was analyzed by blind molecular docking.

#### 4.9.1. Ligand Preparation

The structures of the ligands were geometrically optimized using Avogadro (Software version 1.2.0 https://avogadro.cc/ accessed on 20 June 2022). The possible ionization state was generated at specific pH values (7.0 ± 2.0).

#### 4.9.2. Protein Structure Preparation

The amino acid sequences of CYP450 and NO were obtained from the Protein Data Bank of RCSB. The structure of CYP450 corresponds to PDB: 1OG5 (structure of human cytochrome P450 CYP2C9) with 475 amino acid residues and the structure of NO corresponds to PDB: 2CDU (structure of *Fructilactobacillus sanfranciscensis* water-forming NAD(P)H oxidase) with 452 residues. The protein structures were refined using USCF Chimera software version 1.15 (https://www.cgl.ucsf.edu/chimera, accessed on 20 June 2022). For this purpose, excess solvents, ions, and individual amino acid residues were removed, and the obtained structures were minimized by Swiss PDB 4.10 software Swiss PDB 4.10 (https://spdbv.unil.ch/ accessed on 20 June 2022) [62] After the preparation of the proteins and ligand structures, molecular docking calculations were performed using the SwissDock server (http://www.swissdock.ch/) [63], all databases were accessed on 20 June 2022.

These docking studies corresponded to a rigid system. The best protein–ligand binding models were obtained using specific scoring functions based on energy terms. Interaction types and distances were evaluated using USCF Chimera Discovery Studio Visualizer software, CA, USA (https://www.3ds.com/) accessed on 20 June 2022 [63].

### 4.10. PBPK Model Building

The PBPK analysis was performed with PK-Sim Version 10, which is part of the Open Systems Pharmacology Suite (www.open-systems-pharmacology.org, accessed on 20 June 2022) [13]. Physico–chemistry properties of 21 NP, 14 NP, 6 PP, 20 NP, and 22 NP were accomplished using pK Sim, a freely accessible web server (http://structure. bioc.cam.ac.uk/pkcsm) accessed on 20 June 2022 [64]. Molecule structures and pKa values of molecules were drawn and predicted into Marvin Sketch V22.11 (Marvin|ChemAxon 2022) Marvin (https://chemaxon.com/marvin), and Marvin 17.21.0, Chemaxon (https://www.chemaxon.com), all databases were accessed on 20 June 2022. 

The general workflow of the PBPK model was performed as follows. A PBPK model was developed for a healthy male individual using a Mexican American-White male individual (70 years of age, with a body weight of 74.43 Kg and a height of 165.96 cm [65]). After that, drug-specific parameters for ADMET properties (e.g., lipophilicity) were predicted using pkCSM [64] using the chemical structure of 21 NP, 14 NP, 6 APP, 20 NP, and 22 NP, sketched on MarvinSketch (Chemaxon, Hungary).

## 5. Conclusions

We found that rosmarinic acid, chrysin, naringenin, and cordycepin were the most connected compounds on our network pharmacological analysis. From fingerprinting, we create a novel library of compounds from which we obtain five derived compounds that have higher MAO-B affinity and lead-likeness potential, from such we found that 6 APP may be the most active compound (MAO-B, NO, and CYP450) and may be subjected experimental test against PD.

## Figures and Tables

**Table 1:** Docking test for evolutionary library compounds against the MAO-B.

	MAO-B		
Compounds	Full Fitness (kcal/mol)	Estimated ΔG (kcal/mol)	Binding Energy (kcal/mol)
Selegiline (MAOB-I)	−2242.3184	−6.79202	−12.4572
Rasagiline (MAOB-I)	−2254.627	−6.8181977	−12.2604
Cordycepin-derived compound 21 NP (**1**)	−2293.7556	−9.754256	−13.6346
Rosmarinic acid-derived compound 14 NP (**2**)	−2206.5972	−9.025398	5.34911
Rosmarinic acid-derived compound 6 APP (**3**)	−2264.7612	−8.981724	−21.0128
Naringenin-derived compound 20 NP (**4**)	−2259.6467	−8.539444	−17.4789
Chrysin-derived compound 22 NP (**5**)	−2265.2837	−8.242364	−23.0139
Chrysin-derived compound 20 APP (**6**)	−2233.857	−8.174571	1.13213
Rosmarinic acid-derived compound 18 NP (**7**)	−2204.3083	−8.12989	12.598
Chrysin-derived compound 19 NP (**8**)	−2241.1152	−7.9971757	3.67269
Naringenin-derived compound 18 NP (**9**)	−2282.8008	−7.717949	−22.522
Chrysin-derived compound 11 APP (**10**)	−2235.878	−7.5757804	15.3924
Chrysin fragment 16 NP (**11**)	−2230.024	−7.515393	4.66808
Chrysin fragment 20 NP (**12**)	−2268.7068	−7.207463	5.43122
Naringenin fragment 19 APP (**13**)	−2258.6753	−7.1575794	−18.8473
Rosmarinic acid fragment 15 NP (**14**)	−2217.6724	−7.0223274	4.64089
Rosmarinic acid fragment 21 NP (**15**)	−2235.1602	−7.012647	−17.5915
Rosmarinic acid fragment 16 APP (**16**)	−2259.545	−6.911508	−12.4415
Rosmarinic acid-derived compound 19 APP (**17**)	−2237.1956	−6.8166437	12.0367
Naringenin fragment 23 NP (**18**)	−2238.6543	−6.244289	1.68624

**Table 2:** Compound docking test of selected compounds with higher activity against P450 cytochrome and NO oxidase.

	Cytochrome P450		
Compounds	Full Fitness (kcal/mol)	Estimated ΔG (kcal/mol)	Binding Energy (kcal/mol)
Cordycepin-derived compound (21 NP)	−4482.541	−7.754484	−1.887
Rosmarinic acid-derived compound (14 NP)	−4437.125	−8.617973	17.7735
Rosmarinic acid-derived compound (6 APP)	−4468.041	−8.255595	−4.12677
Naringenin-derived compound (20 NP)	−4481.2944	−7.580227	−5.77106
Chrysin-derived compound (22 NP)	−4449.683	−7.7609963	−3.99852
	**NADPH Oxidase (NO)**		
**Compounds**	**Full Fitness (kcal/mol)**	**Estimated ΔG (kcal/mol)**	**Binding Energy (kcal/mol)**
Cordycepin-derived compound (21 NP)	−4495.0576	−8.262831	−4.28877
Rosmarinic acid-derived compound (14 NP)	−4442.3994	−8.091313	21.4074
Rosmarinic acid-derived compound (6 APP)	−4481.2437	−8.725966	−12.686
Naringenin-derived compound (20 NP)	−4494.463	−7.7760835	−16.9568
Chrysin-derived compound (22 NP)	−4459.724	−7.618342	−5.32596

## Data Availability

Not applicable.

## Supplementary Materials

The following supporting information can be downloaded at: https://www.mdpi.com/article/10.3390/ijms24021134/s1.
