# Supplementary material for: Data-Driven Approaches Used for Compound Library Design for the Treatment of Parkinson’s Disease
Source: Int J Mol Sci. 2023 Jan 6;24(2):1134. doi: 10.3390/ijms24021134 (PMC9867512; doi:10.3390/ijms24021134)
Supplement: Supplementary file 1 [file ijms-24-01134-s001.zip › Supplementary material Tables S2-S5.pdf]

Supplementary material Table S2. Complete data from the PBPK modelling corresponding to 21 NP compound. PK-Analyses of 21NP at 10 mg/Kg concentration obtained by PK-Sim software ver. 11. Important pharmacokinetics terms that describe and quantify aspects of the plasma concentration-time profile of an administered drug that includes Cmax, Vd and AUC.

21NP Molecule

Compartment	Parameter		
	Concentration max (µmol/L)	Area under the curve (µmol*min/L)	Volume of distribution (ml/kg)
Peripheral Venous Blood Plasma Concentration	50.98496628	4902.458496	13070.41931
Brain Tissue	9.761239052	10919.19434	-
Brain Intracellular	9.797018051	10950.96875	-

Supplementary material Table S3. Complete data from the PBPK modelling corresponding to 21 NP compound. PK-Analyses of 21NP at 100 mg/Kg concentration obtained by PK-Sim software ver. 11. Important pharmacokinetics terms that describe and quantify aspects of the plasma concentration-time profile of an administered drug that includes Cmax, Vd and AUC.

21NP Molecule

Compartment	Parameter		
	Concentration max (µmol/L)	Area under the curve (µmol*min/L)	Volume of distribution (ml/kg)
Peripheral Venous Blood Plasma Concentration	509.849823	49024.64844	13071.24043
Brain Tissue	97.61241913	109191.9297	-
Brain Intracellular	97.97020721	109509.6719	-

Supplementary material Table S4. Complete data from the PBPK modelling corresponding to 6PP compound. PK-Analyses of 6PP molecule at 10 mg/Kg concentration obtained by PK-Sim software ver. 11. Important pharmacokinetics terms that describe and quantify aspects of the plasma concentration-time profile of an administered drug that includes Cmax, Vd and AUC.

6PP Molecule

Compartment	Parameter		
	Concentration max (µmol/L)	Area under the curve (µmol*min/L)	Volume of distribution (ml/kg)
Peripheral Venous Blood Plasma Concentration	56.51828384	4696.896973	12652.68898
Brain Tissue	5.816468239	6366.901367	-
Brain Intracellular	5.837036133	6382.829102	-

Supplementary material Table S5. Complete data from the PBPK modelling corresponding to 6PP compound. PK-Analyses of 6PP at 100 mg/Kg concentration obtained by PK-Sim software ver. 11. Important pharmacokinetics terms that describe and quantify aspects of the plasma concentration-time profile of an administered drug that includes Cmax, Vd and AUC.

6PP Molecule

Compartment	Parameter		
	Concentration max (µmol/L)	Area under the curve (µmol*min/L)	Volume of distribution (ml/kg)
Peripheral Venous Blood Plasma Concentration	46968.96875	565.1828613	12652.78149
Brain Tissue	63669.01563	58.16467667	-
Brain Intracellular	63828.29297	58.37035751	-
